# The impact of blood shear rate on arterial thrombus formation

**DOI:** 10.4155/fso.15.28

**Published:** 2015-11-01

**Authors:** Kjell S Sakariassen, Lars Orning, Vincent T Turitto

**Affiliations:** 1 KellSa s.a.s., Str. Campo e Zampo 12, I-13900 Biella, BI, Italy; 2 Alere Technologies AS, POB 6863 Rodelokka, N-0504 Oslo, Norway; 3 Illinois Institute of Technology, 3440 S. Dearborn Street, Chicago, IL 60616, USA

**Keywords:** arterial stenosis, platelet activation, platelet adhesion, platelet aggregation, shear rate, shear stress

## Abstract

The shear rate and corresponding shear stress have impacts on arterial thrombus formation. In particular, the effects of increasing concentration of platelets at the vessel wall and activation of platelets at this site increase the growth and stability of the thrombi which may result in a fatal narrowing of the arterial lumen. The efficacy of many antithrombotic agents is shear dependent as well. It is apparent that there is a need for a point-of-care device to rapidly monitor the risk for arterial thrombosis and to optimize antithrombotic therapy *in vitro.* The present review focuses on the essential role of shear rate on arterial thrombus formation in native human blood drawn directly from an antecubital vein.

## Abstract (lay abstract)

Arterial thrombosis formation is to a large extent determined by the physical parameters of arterial blood flow, that is, shear rate and shear stress. In addition, the efficacy of many antithrombotic agents is dependent upon the shear. Consequently, there is a medical need for a rapid global assay to assess prothrombotic risk and antithrombotic treatment that is performed under arterial blood flow conditions, using native blood, which has not been *in vitro* anticoagulated.

**Figure 1.**
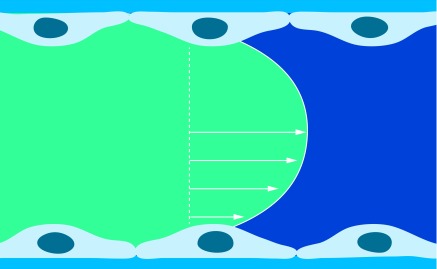
**Artery parabolic blood flow velocity profile.** Velocity is maximal in the central part of the vessel and gradually diminishes as blood approaches the vessel wall.

**Figure 2.**
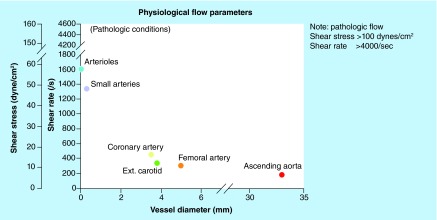
**Vessel wall shear rates and corresponding shear stresses under normal physiological and pathological (stenosis) conditions.**

**Figure 3.**
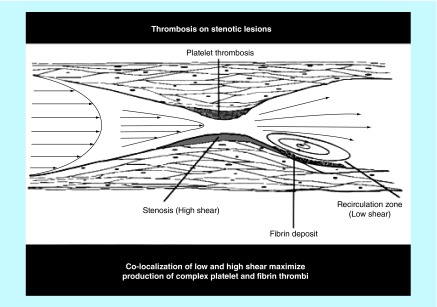
**Blood flow properties and thrombus formation at an atherosclerotic/stenotic lesion.**

**Figure 4.**
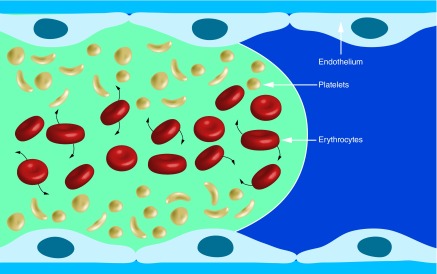
**Distribution of platelets and erythrocytes in arterial flowing laminar blood.** The rotating and deforming erythrocytes increase the concentration of platelets at the vessel wall.

**Figure 5.**
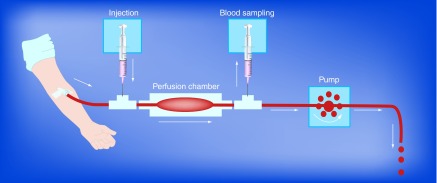
**Human *ex vivo* parallel-plate blood perfusion device.** Blood is drawn from an antecubital vein by the pump. *Ex vivo* studies of therapeutic agents not requiring *in vivo* metabolism can be injected upstream to the perfusion chamber with the reactive surface. Blood sampling for biomarker analysis can be collected downstream to the perfusion chamber. Reproduced with permission from [19] © Future Medicine (2007).

**Figure 6.**
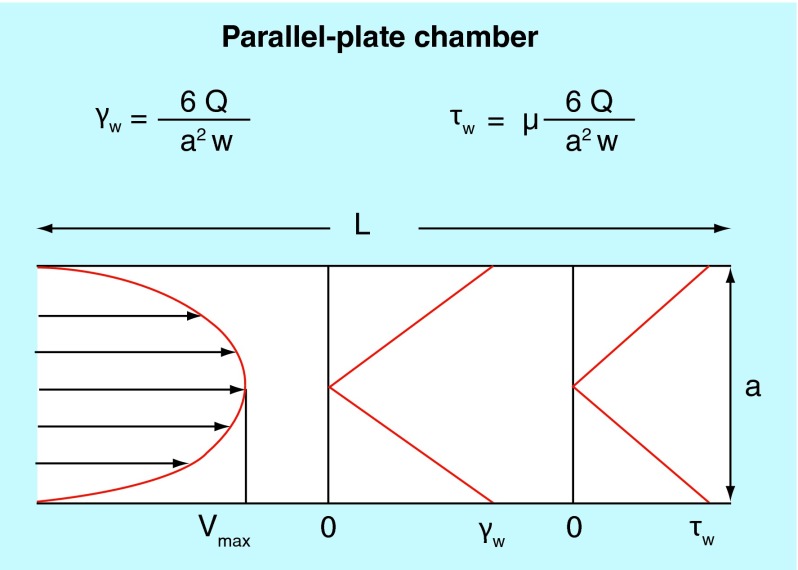
**Typical blood flow velocity profile in the parallel-plate blood perfusion chamber.** The shear rate and shear stress profiles would basically be related to the slope of the velocity profile – zero at center and maximal at the wall where the velocity is zero. Formulas for calculation of wall shear rate (Y) and shear stress (u) are indicated above the figure.

## Identification of physical parameters of blood flow, shear rate & shear stress

The physical blood flow parameters, shear rate and shear stress were identified in the early 1970's and subsequently investigated for their potential impact on arterial thrombus formation [1–5]. Fully developed laminar blood flow in circular tubes, parallel-plate chambers or vessels can be characterized with a parabolic flow velocity profile, with velocity being maximal in the central part of the vessel and gradually diminishing as blood approaches the vessel wall, Figure 1. Correspondingly, the shear rate at the wall (termed the wall shear rate) is maximal where the flow gradient is greatest and the flow velocity is zero. For normal vascular flow, narrowing of the arterial diameter (stenosis) while maintaining blood flow rate constantly increases the wall shear rates and shear stresses that depend on the extent of reduction of the vessel lumen (in a manner that is inversely proportional to the cube of the vessel diameter, 1/d3). This effect is demonstrated physiologically by the highest physiological shear rates and shear stresses being present in the smallest vessels. The strong dependence on wall shear rate and stresses on diameter, results in values of wall shear rate and shear stress in the presence of severe atherosclerotic arteries and at the surface of atherosclerotic plaques, become pathological and increase by one or two orders of magnitude over normal arterial shear conditions, Figure 2. The lowest values of wall shear rates and shear stresses are generally present in the larger vessels of the venous circulation.

The shear rate is defined as the rate of increase of blood flow velocity of adjacent streaming layers (the slope of the velocity profile). The maximum value of the shear rate is located at the vessel wall and can be calculated as follows:
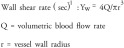



The shear stress which is defined as the force per unit area on the vessel wall can be calculated as follows:
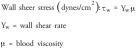



The gradient in the blood flow speed (slope of the velocity profile) in the laminar layers is highest at the vessel wall (Figure 1). This shear rate is termed wall shear rate. Under normal physiological flow conditions, the wall shear rate increases from about 10 s^-1^ in veins to about 2000 s^-1^ in the smallest arteries, whereas maximal wall shear rates up to 40,000 s^-1^ have been described for severe atherosclerotic arteries (Figure 2) [6,7]. The corresponding wall shear stresses are typically 0.35–70 dynes/cm^2^ in the normal vasculature to above 1500 dynes/cm^2^ in diseased vessels depending on the degree of stenosis, as follows from the above equation with the blood flow viscosity assumed to be 0.035 centipoise (g/cm-s). It should be noted that such calculations are order of magnitude estimates for the specific shear conditions, especially in the sclerotic vessels where conditions are complex and computational methods have to be used to determine the actual distribution of shear conditions. For extensive stenosed conditions, the blood flow immediately distal to a severe atherosclerotic lesion may be significantly disturbed and possibly turbulent, promoting the formation of a fibrin-rich thrombus (Figure 3) [8].

Increasing shear rate results in an increase of erythrocytes in the central blood flow layers, which promotes a corresponding increase of platelets close to the vessel wall (Figure 4) [9]. In addition, erythrocytes, which constitute 40% of the blood volume are deforming and rotating. The complex interactions of these cells and their tendency to migrate away from the vessel wall due in part to lift forces induced by flow gradients promotes platelets to be transported toward the vessel wall, and results in an increase of the platelet count near the vessel wall [9–11]. This high concentration of platelets promoted in part by high wall shear rate and corresponding high wall shear stress contribute significantly to the rate and extent of arterial thrombus formation.

## Device & method to study shear rate dependent arterial thrombus formation

Shear rate dependent arterial thrombus formation was studied in a parallel-plate blood perfusion chamber device that draws human blood directly from an antecubital vein (Figure 5) [12,13]. The wall shear rates were experimentally determined and both wall shear rates and shear stresses can be theoretically calculated as well (Figure 6) [14]. Neither platelets nor coagulation are activated in the native blood drawn from the antecubital vein into the perfusion chamber. A broad range of prothrombotic surfaces can be employed in the perfusion chamber, for example, human type III collagen fibrils, human tissue factor plus phospholipids and human stimulated endothelial cells expressing tissue factors [15–18].

Thrombus formation is assayed following 3–5 min of perfusion by computer-assisted light microscopy at 1000× magnification of 1 um thick epon embedded sections. The thrombotic deposits are classified as platelet adhesion (% of surface covered by platelets), thrombus volume (µm3/µm2 surface) and fibrin deposition (% of surface covered by fibrin) [20]. In addition, platelet deposition is further assayed by determining the levels of P-selectin or β-TG as number of platelets/cm^2^ and fibrin deposition is assayed by d-dimer as µg fibrin/cm^2^ following plasmin digestion of the thrombotic deposits [17,21–22]. The blood flow rate employed typically is 10 ml/min with the corresponding wall shear rates varying from 100 to 32.000 s^-1^ depending on the dimensions of the perfusion chamber blood flow channels and on the absence or presence of a stenosis in the flow channels (Table 1) [14,22–23].

## Impact of shear rate on arterial thrombus formation

The shear rate has a profound effect on the interactions of the platelets with the surface in the process of arterial thrombus formation. Platelet deposition is increased in parallel with the increasing shear rate [15,22–24]. The increase in shear rate both activates platelets and promotes their transport to and on and around the growing thrombus, resulting in both increased platelet adhesion and cohesion [15,24]. The combined increase of the near wall platelet numbers and platelet activation by high shear rates and shear stresses, respectively increases the thrombus growth in parallel [23,25]. Typically, these arterial thrombi are rich in platelets and have significantly less fibrin than venous thrombi, and are characteristic of the white thrombus found in arteries. Key platelet receptors for adhesion and cohesion are GPIb and GPIIb–IIIa [26,27]. The coagulation protein thrombin is a strong activator of platelets and their cohesion, and is also a key protein in the formation of fibrin which stabilizes the growth of mural thrombi and physically resists the flow forces due to shear stress and the blood pressure [13]. The effect of the shear rate on arterial thrombus formation with regard to various platelet receptors and coagulation factors was studied with the parallel-plate blood perfusion chamber device with human type III collagen fibrils as prothrombotic surface (Figure 5) [12–15]. The following hereditary bleeding disorders were studied: severe deficiency of vWF, known as von Willebrand disease (vWD subtype III), the vWD subtypes vWD I, vWD IIa, vWD IIb and vWD 2M, deficiency of the platelet receptors GPIa, GPIb and GPIIb–IIIa, and polymorphisms of GPIa, GPIb and GPIIb–IIIa. It is apparent that the deposition of platelets on surfaces exposed to many deficiencies/abnormalities of vWF and platelet receptors is highly shear rate dependent, both with regard to platelet adhesion and cohesion. Increasing shear rate decreased the platelet cohesion, resulting in a gradual decrease in thrombus formation. This was the case for the vWF deficiency and the vWD subtypes investigated [28,29]. Polymorphism GPIa (*C807T*) increased platelet cohesion at high arterial shear rate [30]. Polymorphism of GPIb (*VNTR*) had no effect on thrombus formation, whereas the GPIb (*Kozak type*) increased platelet cohesion at low arterial shear rate [30]. GPIIb–IIIa deficiency reduced both platelet cohesion and fibrin deposition [12].

The shear rate dependence of thrombus formation associated with deficiencies of the coagulation factors FVII and FVIII was studied on human type III collagen fibrils. Fibrin deposition was decreased at all shear rates tested [28,31]. Reduced platelet cohesion was present at low shear rates with low plasma levels of FVIII, whereas reduced platelet cohesion was observed at high shear rate with low plasma levels of FVII.

It is apparent that the deficiency and various vWD subtypes and the deficiency and polymorphism of the studied platelet receptors and coagulation proteins affect the mechanisms of thrombus formation in a somewhat contrasting manner which appears largely dependent on the local wall shear rate. The impact of the tested platelet receptors appear best demonstrated at high arterial shear rate, whereas the low level plasma coagulation factors tested appear at both high and low arterial shear rates. It should be emphasized that the chemical nature of the thrombogenic surface, whether procoagulant or not, may affect the impact of low plasma levels of FVII and FVIII on thrombus formation.

It should also be mentioned that nonlaminar turbulent-like blood flow as present immediately downstream to a severe atherosclerotic lesion promotes in particular coagulation and deposition of fibrin, presumably due to the low shear conditions there and the preexposure to the upstream reactive stenosis, Figure 3. The propagation of this activation of the coagulation system result in the creation of a fibrin-rich tail of the thrombus [32]. The impact of such blood flow conditions on the studied platelet receptors and low level of plasma coagulation factors has not yet been studied.

## Impact of shear rate on antiplatelet agents on arterial thrombus formation

A broad spectrum of antiplatelet agents at optimal clinical doses have been investigated and compared at various well-controlled wall shear rates by using the parallel-plate blood perfusion device (Figure 5) [12–14]. Purified human type 3 collagen fibrils were generally employed as the reactive surface, although some studies were performed with a human tissue factor/phospholipid surface and a procoagulant cellular matrix of human endothelial cells [15–18].

The thromboxane inhibitor, aspirin, inhibited human collagen induced thrombus formation at arterial shear rates, but lost its antithrombotic efficacy at an arterial wall shear rate of 10,500 s^-1^. The loss of antiplatelet efficacy of aspirin at high arterial shear rates has also been demonstrated in patients with severe atherosclerotic lesions [33]. The ADP receptor antagonists ticlopidine and clopidogrel, and the thromboxane receptor antagonist linotroban all inhibited human collagen induced thrombus formation at all arterial wall shear rates studied [34–36]. The GPIIb–IIIa inhibitors RGDS and Ro 44–9883 inhibited human collagen and human cellular matrix induced arterial thrombus formation as well [17,37]. Clopidogrel at the optimal clinical dose appeared as the most efficient inhibitor of arterial thrombus formation.

Combination therapies of aspirin and ticlopidine, aspirin and clopidogrel and aspirin and dipyridamole enhanced the antithrombotic efficacy of each of the antiplatelet agents [34,38–39]. Dipyridamole is an antiplatelet agent which increases the uptake of adenosine resulting in high levels of cAMP which have been demonstrated to inhibit platelet function [40]. The most potent antithrombotic efficacy was observed with the combinations of aspirin and ticlopidine and aspirin and clopidogrel. In this regard it is interesting to note that a combination of aspirin and clopidogrel is currently used therapeutically to avoid cardiovascular events in acute coronary syndromes and in vascular stent induced thrombosis [41]. Combination of aspirin and dipyridamole is used as a therapeutic approach to avoid secondary stroke events [42].

## Impact of shear rate on anticoagulants on arterial thrombus formation

A number of anticoagulants, including low molecular weight heparin, fraxiparine, the vitamin K antagonists fluinidone and marevan, the thrombin inhibitor PEG-hirudin and inhibitors of FVIIa, tissue factor, tissue factor/FVIIa, thrombin, FXa and FIXa were also investigated with the parallel-plate blood perfusion device in a manner similar to that of the antiplatelet agents (Figure 5) [12–14]. The procoagulant surfaces used in the blood perfusion device were tissue factor/phospholipids, subendothelium containing tissue factor and human type III collagen fibrils [15–17]. In general, it was found that the anticoagulants were most efficient in inhibiting platelet cohesion and fibrin deposition on the procoagulant surfaces and much less on the collagen surface at the various arterial wall shear rates tested [13,17,43–49]. The corresponding fibrin deposition was reduced to a similar extent on the various surfaces.

Other physiological anticoagulants, including tissue factor pathway inhibitor, annexin V, ATRA were tested as well, and found to significantly inhibit fibrin deposition at shear rates below 650 s^-1^ [50–52].

A combination therapy of fluinidone and aspirin was tested at high arterial shear rate (2.600 s^-1^) on both human type III collagen fibrils and tissue factor/phospholipid surfaces. Significant inhibition of platelet cohesion and fibrin deposition was observed on both surfaces, although the inhibition was most pronounced on the procoagulant surface [45].

A more detailed analysis of the data obtained with the parallel-plate blood perfusion device with regard to platelet and coagulation disorders and to the effects of antithrombotics and prohemostatic compounds were previously reported together with data obtained by other blood perfusion devices [4,19,53].

## Conclusions and future perspective

Improved understanding of the factors that influence thrombosis, the conditions under which antithrombotic therapies need to be tested and the eventual development of such therapies that block thrombotic events without affecting hemostasis are sorely needed. Such developments will lead to better assessment of thrombotic risk and its treatment and improved treatment of patient care. The increased interest in chamber-based assays to measure thrombus formation has prompted international recommendations for a standardization of devices, protocols and measurement parameters of flow chambers [54–56].

A point-of-care device to rapidly monitor the risk for arterial thrombosis and to optimize antithrombotic therapy is highly needed. Such a device should assess the thrombotic risk in native blood at various arterial shear rates and avoid the artifacts of *in vitro* anticoagulation.

**Table 1.** **Selected *ex vivo* perfusion chamber models of human thrombosis.**

**Chamber type**	**Wall shear rate (s^-1^) at 10 ml/min**	**Corresponding vessel condition**
Parallel	100	Venous
Parallel	650	Average-sized arteries
Parallel	2600	Diffuse atherosclerosis
Stenosis^†^	2600	Moderate arterial stenosis
Stenosis^†^	10,500	Severe arterial stenosis
Stenosis^†^	32,000	Very severe arterial stenosis

^†^Thrombus formation at the stenosis apex.

**Executive summary**

It is fair to conclude that the impact of the wall shear rate on arterial thrombosis has gained much attention and has resulted in improved understanding of the pathogenesis of thrombotic disorders and its treatment in general.
The increased interest in chamber-based assays to measure thrombus formation has prompted international recommendations for a standardization of devices, protocols and measurement parameters of flow chambers.
Much remains to be explored, particularly regarding improvements needed to determine optimal efficiencies in individual subjects at the level of personalized medicine. This goal may be reached by the development of a shear rate dependent point-of-care global prothrombotic/hemostasis assay making use of blood samples not anticoagulated *in vitro*.
